# Tamoxifen Application Is Associated with Transiently Increased Loss of Hippocampal Neurons following Virus Infection

**DOI:** 10.3390/ijms22168486

**Published:** 2021-08-06

**Authors:** Kirsten Hülskötter, Fred Lühder, Alexander Flügel, Vanessa Herder, Wolfgang Baumgärtner

**Affiliations:** 1 Department of Pathology, University of Veterinary Medicine Hannover, Foundation, 30559 Hannover, Germany; kirsten.huelskoetter@tiho-hannover.de (K.H.); vanessa.herder@tiho-hannover.de (V.H.); 2 Center for Systems Neuroscience, 30559 Hannover, Germany; fluegel@med.uni-goettingen.de; 3 Institute for Neuroimmunology and Multiple Sclerosis Research, University Medical Center Göttingen, 37075 Göttingen, Germany; fred.luehder@med.uni-goettingen.de

**Keywords:** tamoxifen, C57BL/6, TMEV, hippocampus, astrocytes, CreER/LoxP

## Abstract

Tamoxifen is frequently used in murine knockout systems with CreER/LoxP. Besides possible neuroprotective effects, tamoxifen is described as having a negative impact on adult neurogenesis. The present study investigated the effect of a high-dose tamoxifen application on Theiler’s murine encephalomyelitis virus (TMEV)-induced hippocampal damage. Two weeks after TMEV infection, 42% of the untreated TMEV-infected mice were affected by marked inflammation with neuronal loss, whereas 58% exhibited minor inflammation without neuronal loss. Irrespective of the presence of neuronal loss, untreated mice lacked TMEV antigen expression within the hippocampus at 14 days post-infection (dpi). Interestingly, tamoxifen application 0, 2 and 4, or 5, 7 and 9 dpi decelerated virus elimination and markedly increased neuronal loss to 94%, associated with increased reactive astrogliosis at 14 dpi. T cell infiltration, microgliosis and expression of water channels were similar within the inflammatory lesions, regardless of tamoxifen application. Applied at 0, 2 and 4 dpi, tamoxifen had a negative impact on the number of doublecortin (DCX)-positive cells within the dentate gyrus (DG) at 14 dpi, without a long-lasting effect on neuronal loss at 147 dpi. Thus, tamoxifen application during a TMEV infection is associated with transiently increased neuronal loss in the hippocampus, increased reactive astrogliosis and decreased neurogenesis in the DG.

## 1. Introduction

Tamoxifen, a selective estrogen receptor modulator (SERM), is used as an inductive agent in the CreER/LoxP system, which is commonly applied in genetically modified mice with a C57BL/6 (B6) background [1]. The inserted tamoxifen-regulated fusion protein (CreER) is very sensitive to tamoxifen and its main metabolite 4-hydroxytamoxifen (4-HT) without binding to endogenous estrogens [2]. For efficient and complete induction of the CreER/LoxP system, high doses of around 2–3 mg tamoxifen per repetitive application are required [3]. The optimal dose and number of applications depend on the targeted organ and delivery method, e.g., oral application or intraperitoneal injection [3]. Conversely, tamoxifen does not exclusively bind to CreER but also to endogenous estrogen receptors and has immunomodulatory properties on various cell types involved in antigen presentation and cellular as well as humoral immune response [2]. In terms of neurological implications, tamoxifen has positive effects on learning abilities and memory [4], cell density [5] and protection against neurotoxin-induced cell death in the hippocampus [6]. Less well-investigated is the negative effect of tamoxifen on prenatal and adult neurogenesis in the rodent hippocampus by dysregulation of the Wnt-signaling pathway [7]. The clinical course of an intracranial Theiler’s murine encephalomyelitis virus (TMEV) infection is highly variable and depends both on the virus strain as well as on the host mouse strain [8]. These factors largely determine whether animals show seizures and eliminate the virus from the central nervous system (CNS) [9,10]. Infection with low virulent strains of TMEV is known to have differential effects on the neurogenesis in the dentate gyrus and activation of microglia depending on the susceptibility of the mouse strain [11]. Intracerebral infection of TMEV in resistant B6 mice with the low virulent BeAn-strain results in acute encephalitis with infection and damage to hippocampal neurons prior to the elimination of the virus, without affecting the neurogenesis [8].

In the present study, a dosage of 3 mg per application was administered every second day for 5 days was used, which would be sufficient to induce a knockout of CD28 in the used mouse strain under the condition of Cre-expression [1]. Furthermore, the effect of tamoxifen treatment on virus elimination, T cell infiltration, hippocampal damage, neurogenesis and glial activation after TMEV infection of genetically modified floxed mice on a B6 background were investigated.

## 2. Short Experimental Setup

All mice were bred on a C57BL/6 (B6) background with floxed CD28 (CD28-/flox) and were infected intracranially with a dose of 5.4 × 10^5^ plaque-forming units of the TMEV-BeAn strain. One group received no tamoxifen (NoTam) and was composed of six mice expressing CreER (CD28-/flox CreER+/−) and six mice without CreER expression (CD28-/flox CreER−/−). The other groups without CreER expression (CD28-/flox CreER−/−) received an oral gavage of 3 mg tamoxifen in rapeseed oil at days 0, 2 and 4 (Tam0dpi, six mice) or 5, 7 and 9 (Tam5dpi, 10 mice) after TMEV infection (Table 1). The dosage and application scheme were chosen to be applicable for induction of CD28 knockout in CreER-expressing mice [1]. The mice were necropsied 7, 14 or 147 days post infection (dpi).

## 3. Results and Discussion

**Figure 1:**
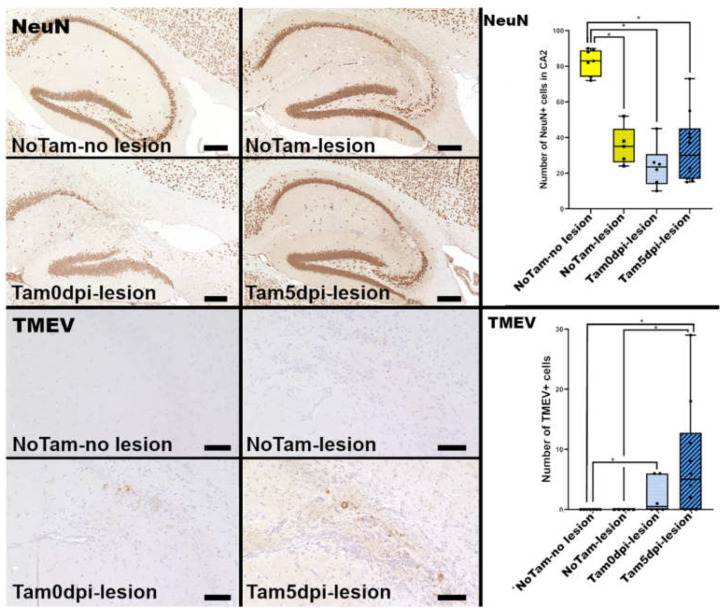
Immunohistochemical evaluation of hippocampi of Theiler’s murine encephalomyelitis virus BeAn (TMEV-BeAn) infected mice 14 days post-infection (dpi) and representative images of each marker, depicting the most prominent findings and statistical analysis of the obtained data on the right. Mice without tamoxifen treatment were separated into two groups; animals with no lesions (NoTam—no lesions) and mice with hippocampal neuronal loss (NoTam—lesions), and mice with tamoxifen treatment 0, 2 and 4 dpi (Tam0dpi—lesion) and mice with tamoxifen treatment 5, 7 and 9 dpi (Tam5dp—lesion). The graphs depict the groups as follows: NoTam—no lesions: yellow, no filling, NoTam—lesions: yellow/V-pattern, Tam0dpi—lesion: light blue/dotted and Tam5dpi—lesion: blue/striped. Tamoxifen-treated animals showed a more frequent occurrence of neuronal loss, consisting of fewer neuron nuclear antigen (NeuN) positive neurons in the cornu ammonis region 2 (CA2) of the hippocampus. Mice without tamoxifen application eliminatedTMEV from the hippocampus within 14 days, while 10 of 16 tamoxifen-treated animals (63%) still showed intralesional TMEV antigen in the hippocampus at this time point. Scale bars: 200 µm (NeuN) and 50 µm (TMEV). Statistical significant differences are depicted as * = *p* ≤ 0.05 (Supplementary Table S1 for each *p*-value individually).

The most prominent feature after TMEV infection of B6 mice was hippocampal neuronal loss within the first 2 weeks. Evaluation of the number of neuron nuclear antigen (NeuN) expressing neurons showed that in this experiment, 5 of 12 mice (42%) without tamoxifen application (NoTam) developed hippocampal inflammatory lesions with neuronal loss at 14 dpi. Thus, this group (Table 1) was divided into mice with neuronal loss (NoTam—lesion, five mice) and mice without loss of pyramidal neurons in the hippocampus (NoTam—no lesion, seven mice). It is commonly seen that hippocampal neuronal loss after TMEV-BeAn infection varies between not detectable and 20–50% loss of the pyramidal neurons in the CA1/CA2 sector in mice on a C57BL/6 background at 7 and 98 dpi [10]. Strikingly, 15 of 16 mice (94%) with tamoxifen application, either starting at 0 dpi (Tam0dpi—lesion, six of six mice) or 5 dpi (Tam5dpi—lesion, 9 of 10 mice), showed prominent neuronal loss in the pyramidal layer of the cornu ammonis (CA) at 14 dpi. Neuronal loss was most prominent in CA2 but also extended into CA1 and CA3 (Figure 1). Thus, the neuroprotective properties of tamoxifen, observed in previous non-infectious studies of the hippocampus [4,5,6], were not corroborated in the present infectious study. Yet, it seems the neuroprotective effect is not present in infectious processes as documented in the present investigation.

Since the loss of hippocampal neurons is associated with their infection [8], viral load and duration of infection can contribute to lesion size. For effective elimination of TMEV from the brain, B6 mice depend on a quick and effective immune response [8], whereas irrespective of lesions, NoTam mice were able to eliminate the virus within 14 days, there was still intralesional virus antigen detectable in Tam0dpi and Tam5dpi mice (Figure 1). The presented results demonstrated that tamoxifen decelerated viral clearance from the hippocampus. Additional experiments in which mice were sacrificed at 7 dpi showed that there is no difference in the numbers of TMEV antigen-positive cells in the hippocampus of Tam0dpi mice at this earlier time point (Supplementary Figure S1).

**Figure 2:**
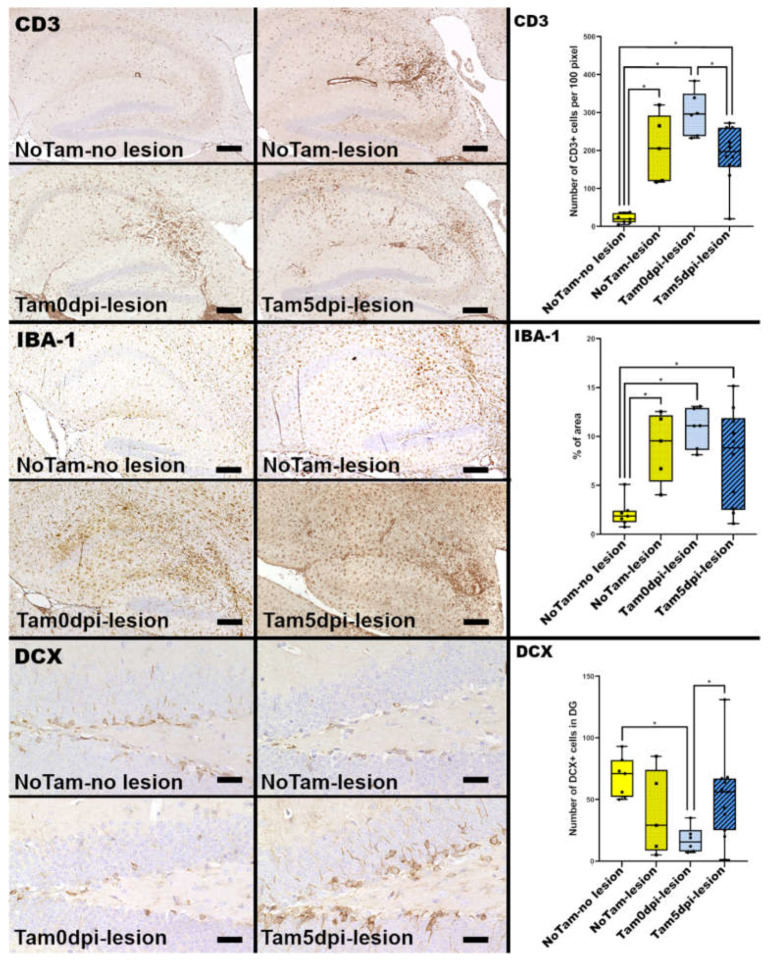
Immunohistochemical evaluation of hippocampi of Theiler’s murine encephalomyelitis virus BeAn (TMEV-BeAn) infected mice 14 days post-infection (dpi). Presence of neuronal loss (Figure 1) is associated with increased numbers of CD3 positive T cells and activated ionized calcium-binding associated molecule-1 (IBA-1) positive microglia/macrophages. The infiltration of CD3 and IBA-1 positive cells within the hippocampus does not differ between tamoxifen-treated and untreated animals without lesions. Tam0dpi mice show decreased numbers of doublecortin (DCX)-positive cells in the DG compared to untreated mice without lesions and Tam5dpi mice. Scale bars: 200 µm (CD3, IBA-1) and 50 µm (DCX). Statistical significant differences are depicted as * = *p* ≤ 0.05 (Supplementary Table S1 for each *p*-value individually).

Impairment of the immune response can interfere with the efficient elimination of TMEV from the murine CNS, as described in previous studies [8]. Tamoxifen is known to modulate the immune response and inhibit T cell activation [2]. However, a detailed analysis of local infiltration of T cells into the hippocampal parenchyma at 14 dpi showed that the number of CD3 positive T cells was elevated in all mice, which developed lesions compared to untreated mice without lesions and was only reduced in the Tam5dpi group compared to Tam0dpi mice (Figure 1). The inhibitory effect of tamoxifen on T cells is considered to be in part mediated by decreased antigen presentation [12]. Therefore, the activation of glial cells was investigated in further detail. In non-infectious models for brain trauma, tamoxifen reduced microglial activation and reactive astrogliosis [13], which, as well as the prompt activation of microglia and macrophages, contributes largely to neuronal loss by bystander damage in B6 mice [8]. Results of the present study indicate that mice with hippocampal neuronal loss also displayed a prominent activation of microglia/macrophages irrespective of tamoxifen treatment, as shown by upregulation of ionized calcium-binding adapter molecule-1 (IBA-1) expression (Figure 2).

In the acute phase at 14 dpi, there was a correlation between the loss of pyramidal neurons and tamoxifen gavage, with a more frequent occurrence of 90–100% in the groups with tamoxifen treatment. To investigate whether this was a long-lasting effect and whether tamoxifen affects neurogenesis, a later time point at 147 dpi was investigated for the extent of neuronal loss. At this time point, the general extent of neuronal loss with 20–21% was lower than at 14 dpi, but notably, this was completely independent of tamoxifen treatment (Supplementary Figure S2). Therefore, similar regeneration can be seen after ischemic damage of the CA1 in mice, and there appears to be a low-level production of new neurons [14]. However, most of the ongoing neurogenesis in the adult hippocampus is restricted to the dentate gyrus (DG) [15], and in the present study, no doublecortin (DCX) positive neuronal progenitors were detected anywhere outside of the DG. TMEV infection alone is not known to have any effect on the number of DCX positive cells in the DG of B6 mice [11]. Despite the positive effects of tamoxifen on neurons after brain injury [4,5,6,16,17], it was seen that even a single dose could negatively impact adult neurogenesis in the DG [7]. Surprisingly, there was a time-dependent effect of tamoxifen on the number of DCX positive neuronal progenitor cells in the DG of the TMEV-infected animals in the present study. Tamoxifen application at the time of TMEV infection (Tam0dpi) was associated with significantly lower numbers of DCX expressing cells at 14 dpi (Figure 1). However, this had no long-term effect on the neuronal loss at 147 dpi (Supplementary Figure S2).

**Figure 3:**
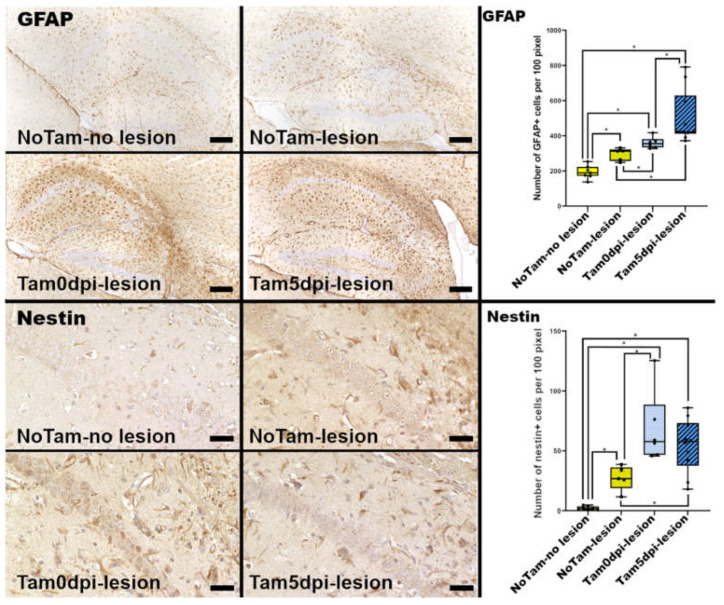
Immunohistochemical evaluation of hippocampi of Theiler’s murine encephalomyelitis virus BeAn (TMEV-BeAn) infected mice 14 days post-infection (dpi). Tamoxifen treatment is associated with astrogliosis characterized by increased numbers of glial fibrillary acidic protein (GFAP)-positive cells in the hippocampus. These nestin-positive cells, most likely representing reactive astrocytes, are similarly increased in tamoxifen-treated animals at 14 dpi. Scale bars: 200 µm (GFAP) and 50 µm (nestin). Statistical significant differences are depicted as * = *p* ≤ 0.05 (Supplementary Table S1 for each *p*-value individually).

Besides microgliosis, astrogliosis was also observed in the hippocampus after TMEV infection [8]. In the present study, it was shown that tamoxifen application, as well as the presence of inflammatory lesions, resulted in increased numbers of glial fibrillary acidic protein (GFAP)-positive as well as nestin-positive cells within the hippocampus at 14 dpi (Figure 3). Nestin and GFAP are co-expressed by radial glia-like stem cells, which differentiate over intermediate progenitor cells (DCX positive) into post-mitotic NeuN positive neurons [18]. Nonetheless, rapid re-expression of nestin is also reported for reactive astrocytes after brain injury in rodents [19]. In the present study, the starry morphology and localization of the nestin-positive cells together with no apparent DCX expression apart from the DG suggest a reactive astrocytic phenotype [20], rather than prominent neurogenesis in the CA (Figure 3).

**Figure 4:**
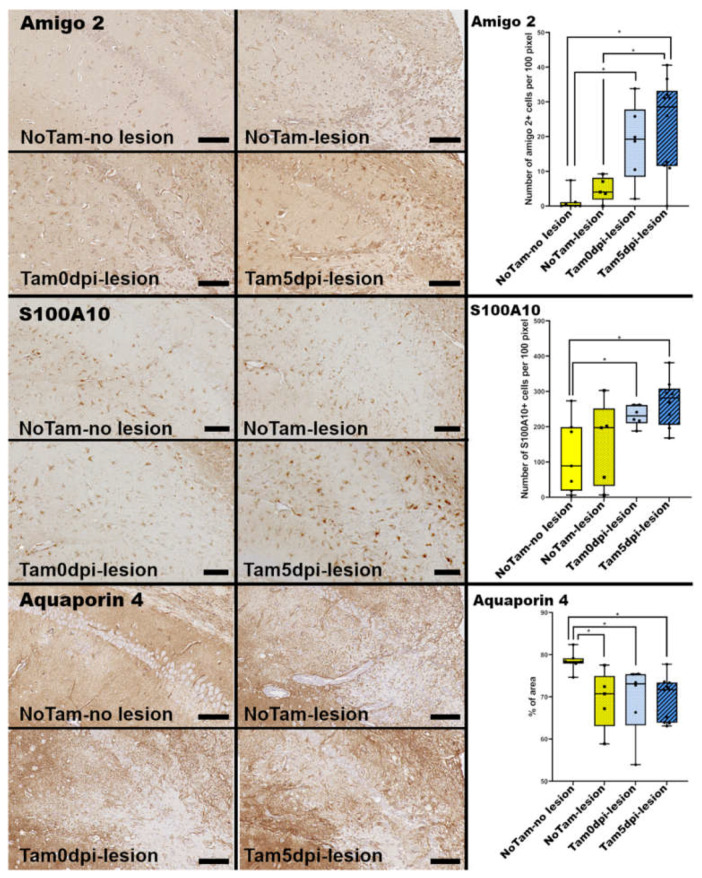
Immunohistochemical evaluation of hippocampi of Theiler’s murine encephalomyelitis virus BeAn (TMEV-BeAn) infected mice 14 days post-infection (dpi). Further differentiation of the astrocyte phenotypes into amigo 2-positive cytotoxic A1 astrocytes and S100A10-positive neurotrophic A2 astrocytes revealed an increase of both phenotypes in tamoxifen-treated animals. The number of cytotoxic A1 astrocytes was higher in tamoxifen-treated animals compared to untreated animals with hippocampal neuronal loss. Aquaporin 4 expression was downregulated in the presence of inflammation but was not further impacted by tamoxifen application. Scale bars: 100 µm (Amigo 2, S100A10, Aquaporin 4). Statistical significant differences are depicted as * = *p* ≤ 0.05 (Supplementary Table S1 for each *p*-value individually).

Reactive astrocytes can be further divided into neurotoxic A1 (amigo 2-positive) and neurotrophic A2 (S100A10-positive) astrocytes [21]. The present results show that the number of cytotoxic A1 astrocytes is significantly elevated in tamoxifen-treated animals, with the strongest effect seen in the Tam5dpi group (Figure 4). A1 astrocyte differentiation is promoted by pro-inflammatory cytokines such as interleukin-1 (IL-1) and tumor necrosis factor-α (TNF-α) [21]. Their transcription is commonly upregulated in response to a TMEV infection [8]. Thus, the prolonged infection of hippocampal neurons in tamoxifen-treated mice might contribute to the increased numbers of A1 astrocytes observed in the hippocampi of these animals in the present study. Tamoxifen-treated animals also showed increased numbers of neurotropic A2 astrocytes compared to untreated mice with no lesions (Figure 4). Aquaporin 4 is the main water channel protein of astrocytes within the CNS, with a high density at the glia limitans [22]. The expression can be negatively impacted by inflammation [22]. In the present study, it was seen that the expression of aquaporin 4 is downregulated within inflammatory hippocampal lesions after TMEV infection, irrespective of tamoxifen application (Figure 4). In summary, tamoxifen gavage is not only associated with more frequent occurrence of neuronal loss but also with increased astrogliosis and higher numbers of reactive astrocytes of both phenotypes within the hippocampus of TMEV-infected B6 mice at 14 dpi.

In general, the impact of tamoxifen was dependent on the time point of application, with the strongest effects detected in mice with tamoxifen application 5, 7 and 9 dpi (Tam5dpi). An explanation why there was a time-dependent effect of tamoxifen might be the increasing leakage of the blood–brain barrier (BBB) during acute TMEV infection with suspected increased penetrance for tamoxifen in the Tam5dpi mice [23]. Tamoxifen gavage had a negative effect on the preservation of hippocampal neurons within the CA during acute TMEV infection, which was most likely due to inhibition of efficient viral clearance and concomitant longer-lasting activation of glial cells. Although tamoxifen did not have a direct impact on all analyzed cell types, the occurrence of inflammatory hippocampal lesions was in general elevated in mice with tamoxifen application (* *p* = 0.003). However, there was no long-term effect of tamoxifen gavage on the persistence of neuronal loss at 147 dpi (*p* = 0.8).

Conclusively, high-dose tamoxifen application during the early phase of a TMEV infection has a transient negative impact on virus elimination from the hippocampus, which is accompanied by increased inflammation and concomitantly increased neuronal loss within the CA. Astrocytes appear to be directly influenced by tamoxifen with a dominant proliferation of neurotoxic A1 reactive astrocytes. The effect of tamoxifen on adult neurogenesis remains inconclusive and requires further investigation. Leakage of the BBB during acute encephalitis might result in increased permeability for tamoxifen and its metabolites and thus might trigger the observed time-dependent effects of tamoxifen gavage. Further studies on parenchymal concentrations of the compound itself and its metabolites in the CNS could reveal changes in the pharmacokinetics of tamoxifen under inflammatory conditions. Furthermore, FACS analysis of CNS tissue should be included in future studies to provide a deeper insight into the effect of tamoxifen on the phenotype and activation status of infiltrating T cells. The presented results show that tamoxifen gavage has a direct and significant impact on the course and lesion development during infectious CNS diseases by influencing the immune response. Since a significant dose reduction is often not feasible to induce complete knockouts in the CreEr/LoxP system [3], possible effects of the tamoxifen gavage on the lesion development should be considered in infectious CNS disease processes.

## 4. Materials and Methods

### 4.1. Mice

FEC2/FLCK CD28KO mice (CD28-/lox CreER+/− and CD28-/lox CreER−/−) on a C57BL/6 background were bred at the animal facility of the Institute for Multiple Sclerosis Research (IMSF), University of Göttingen, Göttingen, Germany [24].

### 4.2. TMEV Infection, Tamoxifen Application and Necropsy

Thirty-five-day-old mice were infected intracerebrally with a dose of 5.4 × 105 plaque-forming units (PFU) of the TMEV-BeAn strain. Tamoxifen was applied by oral gavage of 3 mg tamoxifen diluted in 100 µL rapeseed oil using disposable, flexible 20 G polypropylene feeding tubes for rodents (Instech Laboratories, Inc., Plymouth Meeting, PA, USA, cat. FTP-20-38-50) either at day 0, 2 and 4 or 5, 7 and 9 after infection. At 7, 14 or 147 days after infection, the mice were sacrificed and post-mortally perfused with phosphate-buffered saline (PBS). The brain was fixed for 24 h in 10% buffered formalin prior to embedding in paraffin. 

### 4.3. Immunohistochemistry

Immunohistochemistry was performed using the avidin-biotin-peroxidase complex (ABC) method with diaminobenzidine (DAB) labeling as previously described [25]. If necessary, antigen retrieval was performed by boiling in citrate buffer (pH6) in the microwave (MW) for 20 min or incubation with 0.5% Triton-X solution (T). Tissues were blocked with goat serum. The following primary antibodies were used as previously described [23]: polyclonal rabbit-anti-glial fibrillary acidic protein (GFAP) antibody (Agilent Technologies, Inc., Santa Clara, CA, USA, Dako, cat. Z0334, 1:1000), in-house polyclonal rabbit-anti-TMEV antibody (1:2000), monoclonal mouse-anti-neuron nuclear antigen (NeuN) antibody (Merck-Millipore, Inc., Burlington, MA, USA, cat. MAB377, MW, 1:1600), monoclonal mouse-anti-doublecortin (DCX) antibody (Santa-Cruz Biotechnology, Inc., Dallas, TX, USA, cat. Sc-271390, 1:100), polyclonal rabbit-anti-nestin antibody (OriGene Technologies, Inc., Rockville, MD, USA, cat. AP07829PU-N, 1:500), polyclonal rabbit-anti-ionized calcium-binding adapter molecule-1 (IBA-1) antibody (Invitrogen, Thermo Firscher Scientific, Waltham, MA, USA, cat. PA5-27436, 1:2000), polyclonal rabbit-anti-human CD3 (Agilent Technologies, Inc., Santa Clara, CA, USA, Dako, cat. A0452, MW, 1:250), anti-adhesion molecule with Ig-like domain 2–Type 1 astroglial (amigo 2) antibody (Bioss, Inc., Woburn, MA, USA, cat. Bs-11450R, T, 1:200), anti-S100A10—Type 2 astroglial antibody (Bio-Techne GmbH, Wiesbaden, Germany, cat. Ab JF0987, 1:100), anti-aquaporin 4 (Merck-Millipore, Inc., Burlington, MA, USA cat. AB3594, 1:200).

### 4.4. Statistics, Figures and Data Generation:

Statistics and graphs were generated with R-Studio (RStudio, Inc., Boston, MA, USA) and GraphPad Prism (GraphPad Software, Inc., San Diego, CA, USA). Immunohistochemical datasets were evaluated for significant differences using the Mann–Whitney U test as a non-parametric, distribution-free test for small sample sizes. For testing the effects of CreER-expression or tamoxifen gavage on the occurrence of neuronal loss, the Chi2-test was applied. Pictures were taken with a Keyence photomicroscope (BZ9000) at 100-fold magnification. Figures were processed with GIMP (2.10.22). NeuN-, CD3-, S100A10- and GFAP-positive cells were counted, and areas in aquaporin 4, amigo 2 and IBA-1 labeled sections were measured with ImageJ (v1.51q). TMEV-, DCX-, nestin- and amigo 2-positive labeled cells were counted manually. NeuN positive cells were counted in the CA2, TMEV-positive cells were counted in the whole hippocampus (CA and DG), DCX positive cells were counted in the DG. CD3-, GFAP-, nestin-, amigo 2- and S100A10-positive cells were counted in the whole hippocampus and normalized to an area of 100 pixels. IBA-1 and aquaporin 4 were measured as a percentage of the entire area of the hippocampus.

## Figures and Tables

**Table 1:** Summary of the different groups of TMEV infected mice that were sacrificed 14 days post-infection (dpi), divided by tamoxifen treatment and presence of neuronal loss (lesion) in the CA2 of the hippocampus. The NoTam-groups contain CreER+/− as well as CreER−/− mice. There was no difference in the occurrence of hippocampal lesions in untreated mice with or without CreER-expression (*p* = 0.6).

Group Term	Description	14 dpi
NoTam—no lesion	TMEV-infected B6 mice without tamoxifen application and no neuronal loss in the CA2	*N* = 7
NoTam—lesion	TMEV-infected B6 mice without tamoxifen application and neuronal loss in the CA2	*N* = 5
Tam0dpi—lesion	TMEV-infected B6 mice with tamoxifen application 0, 2 and 4 dpi	*N* = 6
Tam5dpi—lesion	TMEV-infected B6 mice with tamoxifen application 5, 7 and 9 dpi	*N* = 10

## Data Availability

The data that support the findings of this study are available from the corresponding author upon reasonable request.

## Supplementary Materials

The following are available online at https://www.mdpi.com/article/10.3390/ijms22168486/s1.

## References

[1] Fröhlich M., Gogishvili T., Langenhorst D., Lühder F., Hünig T. (2016). Interrupting CD28 costimulation before antigen rechallenge affects CD8(+) T-cell expansion and effector functions during secondary response in mice. Eur. J. Immunol..

[2] Behjati S., Frank M.H. (2009). The effects of tamoxifen on immunity. Curr. Med. Chem..

[3] Donocoff R.S., Teteloshvili N., Chung H., Shoulson R., Creusot R.J. (2020). Optimization of Tamoxifen-Induced Cre Activity and Its Effect on Immune Cell Populations. Sci. Rep..

[4] Zabihi H., Hosseini M., Pourganji M., Oryan S., Soukhtanloo M., Niazmand S. (2014). The effects of tamoxifen on learning, memory and brain tissues oxidative damage in ovariectomized and naive female rats. Adv. Biomed. Res..

[5] Silva I., Mello L.E., Freymuller E., Haidar M.A., Baracat E.C. (2000). Estrogen, progestogen and tamoxifen increase synaptic density of the hippocampus of ovariectomized rats. Neurosci. Lett..

[6] Gursoy E., Cardounel A., Al-khlaiwi T., Al-drees A., Kalimi M. (2002). Tamoxifen protects clonal mouse hippocampal (HT-22) cells against neurotoxins-induced cell death. Neurochem. Int..

[7] Lee C.-M., Zhou L., Liu J., Shi J., Geng Y., Liu M., Wang J., Su X., Barad N., Wang J. (2020). Single-cell RNA-seq analysis revealed long-lasting adverse effects of tamoxifen on neurogenesis in prenatal and adult brains. Proc. Natl. Acad. Sci. USA.

[8] Gerhauser I., Hansmann F., Ciurkiewicz M., Löscher W., Beineke A. (2019). Facets of Theiler’s Murine Encephalomyelitis Virus-Induced Diseases: An Update. Int. J. Mol. Sci..

[9] Bröer S., Hage E., Käufer C., Gerhauser I., Anjum M., Li L., Baumgärtner W., Schulz T.F., Löscher W. (2017). Viral mouse models of multiple sclerosis and epilepsy: Marked differences in neuropathogenesis following infection with two naturally occurring variants of Theiler’s virus BeAn strain. Neurobiol. Dis..

[10] Bröer S., Käufer C., Haist V., Li L., Gerhauser I., Anjum M., Bankstahl M., Baumgärtner W., Löscher W. (2016). Brain inflammation, neurodegeneration and seizure development following picornavirus infection markedly differ among virus and mouse strains and substrains. Exp. Neurol..

[11] Jafari M., Haist V., Baumgärtner W., Wagner S., Stein V.M., Tipold A., Wendt H., Potschka H. (2012). Impact of Theiler’s virus infection on hippocampal neuronal progenitor cells: Differential effects in two mouse strains. Neuropathol. Appl. Neurobiol..

[12] Bebo B.F., Dehghani B., Foster S., Kurniawan A., Lopez F.J., Sherman L.S. (2009). Treatment with selective estrogen receptor modulators regulates myelin specific T-cells and suppresses experimental autoimmune encephalomyelitis. Glia.

[13] Baez-Jurado E., Rincón-Benavides M.A., Hidalgo-Lanussa O., Guio-Vega G., Ashraf G.M., Sahebkar A., Echeverria V., Garcia-Segura L.M., Barreto G.E. (2019). Molecular mechanisms involved in the protective actions of Selective Estrogen Receptor Modulators in brain cells. Front. Neuroendocrinol..

[14] Zhao Z., Sun P., Chauhan N., Kaur J., Hill M.D., Papadakis M., Buchan A.M. (2006). Neuroprotection and neurogenesis: Modulation of cornus ammonis 1 neuronal survival after transient forebrain ischemia by prior fimbria-fornix deafferentation. Neuroscience.

[15] Abbott L.C., Nigussie F. (2020). Adult neurogenesis in the mammalian dentate gyrus. Anat. Histol. Embryol..

[16] Guptarak J., Wiktorowicz J.E., Sadygov R.G., Zivadinovic D., Paulucci-Holthauzen A.A., Vergara L., Nesic O. (2014). The cancer drug tamoxifen: A potential therapeutic treatment for spinal cord injury. J. Neurotrauma.

[17] Colon J.M., Torrado A.I., Cajigas A., Santiago J.M., Salgado I.K., Arroyo Y., Miranda J.D. (2016). Tamoxifen administration immediately or 24 Hours after spinal cord injury improves locomotor recovery and reduces secondary damage in female rats. J. Neurotrauma.

[18] Nicola Z., Fabel K., Kempermann G. (2015). Development of the adult neurogenic niche in the hippocampus of mice. Front. Neuroanat..

[19] Schmidt-Kastner R., Humpel C. (2002). Nestin expression persists in astrocytes of organotypic slice cultures from rat cortex. Int. J. Dev. Neurosci..

[20] Cassé F., Richetin K., Toni N. (2018). Astrocytes’ Contribution to Adult Neurogenesis in Physiology and Alzheimer’s Disease. Front. Cell. Neurosci..

[21] Allnoch L., Baumgärtner W., Hansmann F. (2019). Impact of Astrocyte Depletion upon Inflammation and Demyelination in a Murine Animal Model of Multiple Sclerosis. Int. J. Mol. Sci..

[22] Kaneyama T., Takizawa S., Tsugane S., Yanagisawa S., Takeichi N., Ehara T., Ichikawa M., Koh C.-S. (2013). Downregulation of water channel aquaporin-4 in rats with experimental autoimmune encephalomyelitis induced by myelin basic protein. Cell. Immunol..

[23] Omura S., Kawai E., Sato F., Martinez N.E., Minagar A., Al-Kofahi M., Yun J.W., Cvek U., Trutschl M., Alexander J.S. (2018). Theiler’s virus-mediated immunopathology in the CNS and heart: Roles of organ-specific cytokine and lymphatic responses. Front. Immunol..

[24] Gogishvili T., Lühder F., Kirstein F., Nieuwenhuizen N.E., Goebbels S., Beer-Hammer S., Pfeffer K., Reuter S., Taube C., Brombacher F. (2012). Interruption of Cd28-Mediated Costimulation during Allergen Challenge Protects Mice from Allergic Airway Disease. J. Allergy Clin. Immunol..

[25] Hülskötter K., Jin W., Allnoch L., Hansmann F., Schmidtke D., Rohn K., Flügel A., Lühder F., Baumgärtner W., Herder V. (2021). Double-edged effects of tamoxifen-in-oil-gavage on an infectious murine model for multiple sclerosis. Brain Pathol..

