# Supplementary material for: Tamoxifen Application Is Associated with Transiently Increased Loss of Hippocampal Neurons following Virus Infection
Source: Int J Mol Sci. 2021 Aug 6;22(16):8486. doi: 10.3390/ijms22168486 (PMC8395206; doi:10.3390/ijms22168486)
Supplement: Supplementary file 1 [file ijms-22-08486-s001.zip › ijms-1310611-supplementary.pdf]

Supplemental Material:

Brief Report: Tamoxifen application is associated with transiently increased loss of hippocampal neurons following virus infection

Kirsten Hülskötter[1,2], Fred Lühder[3], Alexander Flügel[2,3], Vanessa Herder[1,2], Wolfgang Baumgärtner[1,2,*]

[1] Department of Pathology, University of Veterinary Medicine Hannover, 30559 Hannover, Germany

[2] Center for Systems Neuroscience, Hannover

[3] University Medical Center Göttingen, Institute for Neuroimmunology and Multiple Sclerosis Research, 37075 Göttingen, Germany

*Corresponding author:

Prof. Dr. Wolfgang Baumgärtner, PhD, Dipl. ECVP, Dipl. ACVP hon.
Department of Pathology
University of Veterinary Medicine Hannover
Bünteweg 17
30559 Hannover
Germany
Email: wolfgang.baumgaertner@tiho-hannover.de
Tel.: +49 511 953 8620
Fax: +49 511 953 8675

Supplementary Table S1: statistically significant p-values (Mann-Whitney U test) depicted in figure 1:

**Antibody**	**Group 1**	**n=**	**vs. Group 2**	**n=**	**p-value**
**Amigo 2**	NoTam-no lesion	7	Tam0dpi	6	0.005
**Amigo 2**	NoTam-no lesion	7	Tam5dpi	10	0.004
**Amigo 2**	NoTam-lesion	5	Tam5dpi	10	0.01
**Aquaporin 4**	NoTam-no lesion	7	NoTam-lesion	5	0.005
**Aquaporin 4**	NoTam-no lesion	7	Tam0dpi	6	0.005
**Aquaporin 4**	NoTam-no lesion	7	Tam5dpi	10	0.0002
**CD3**	NoTam-no lesion	7	NoTam-lesion	5	0.003
**CD3**	NoTam-no lesion	7	Tam0dpi	6	0.001
**CD3**	NoTam-no lesion	7	Tam5dpi	10	0.0007
**CD3**	Tam0dpi	6	Tam5dpi	10	0.007
**DCX**	NoTam-no lesion	7	Tam0dpi	6	0.001
**DCX**	Tam0dpi	6	Tam5dpi	10	0.002
**GFAP**	NoTam-no lesion	7	NoTam-lesion	5	0.005
**GFAP**	NoTam-no lesion	7	Tam0dpi	6	0.001
**GFAP**	NoTam-no lesion	7	Tam5dpi	10	0.0001
**GFAP**	NoTam-lesion	5	Tam0dpi	6	0.009
**GFAP**	NoTam-lesion	5	Tam5dpi	10	0.0007
**GFAP**	Tam0dpi	6	Tam5dpi	10	0.001
**IBA-1**	NoTam-no lesion	7	NoTam-lesion	5	0.005
**IBA-1**	NoTam-no lesion	7	Tam0dpi	6	0.001
**IBA-1**	NoTam-no lesion	7	Tam5dpi	10	0.01
**Nestin**	NoTam-no lesion	7	NoTam-lesion	5	0.003
**Nestin**	NoTam-no lesion	7	Tam0dpi	6	0.001
**Nestin**	NoTam-no lesion	7	Tam5dpi	10	0.0001
**Nestin**	NoTam-lesion	5	Tam0dpi	6	0.004
**Nestin**	NoTam-lesion	5	Tam5dpi	10	0.04
**NeuN**	NoTam-no lesion	7	NoTam-lesion	5	0.003
**NeuN**	NoTam-no lesion	7	Tam0dpi	6	0.001
**NeuN**	NoTam-no lesion	7	Tam5dpi	10	0.0002
**NeuN**	NoTam-lesion	5	Tam0dpi	6	0.02
**S100A10**	NoTam-no lesion	7	Tam0dpi	6	0.05
**S100A10**	NoTam-no lesion	7	Tam5dpi	10	0.007
**TMEV**	NoTam-no lesion	7	Tam0dpi	6	0.05
**TMEV**	NoTam-no lesion	7	Tam5dpi	10	0.009
**TMEV**	NoTam-lesion	5	Tam5dpi	10	0.02

ST1: Statistically significant p-values ($p \leq 0.05$) evaluated by Mann-Whitney U test presented in figure 1 of the main manuscript. Further information about the group labeling and composition can be found in table 1 of the main manuscript. Information about the used antibodies for immunohistochemistry can be found in the materials and methods section of the main manuscript. Significant p-values were depicted in the graphs of figure 1 of the main manuscript as asterisk (*).

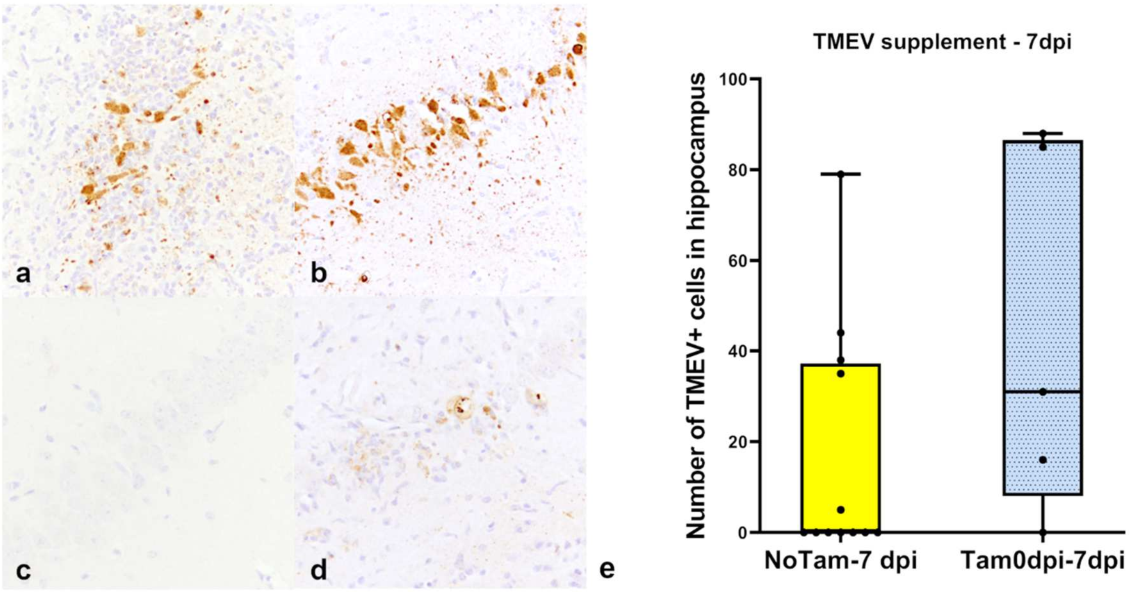

Supplementary figure S1: Hippocampi of TMEV-BeAn infected mice at 7 dpi (a, b) and 14 dpi (c, d). Untreated mice (*NoTam-* a, c) and mice with tamoxifen application 0, 2 and 4 dpi (*Tam0dpi-* b, d) – immunohistochemistry, TMEV 400x magnification. Numerous TMEV antigen positive cells were present in both groups at 7 dpi (a,b) while only few positive cells were detectable in tamoxifen treated mice at 14 dpi (d), compared to untreated mice (c). (e) Number of TMEV antigen positive cells within the hippocampus at 7 dpi comparing untreated mice (*NoTam-* yellow, n=12) and mice with tamoxifen application 0, 2 and 4 dpi (*Tam0dpi-* light blue, dotted, n=5). There is no significant difference between the groups at 7 dpi (p= 0.148) which is in contrast to the findings at 14 dpi (see main manuscript figure 1).

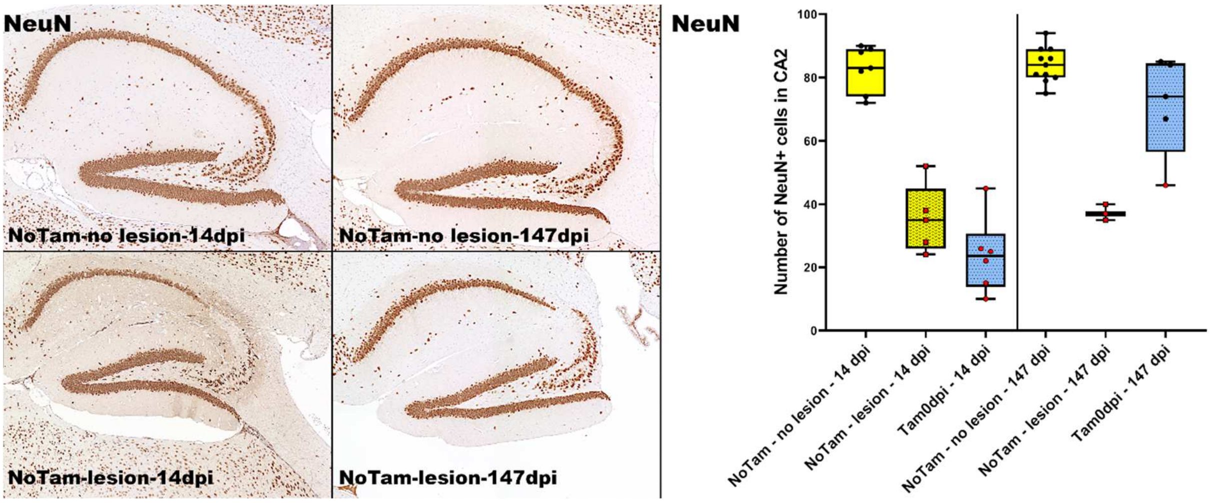

Supplementary figure S2: Hippocampi of untreated, TMEV-infected mice (*NoTam*) without (*no lesion*) and with neuronal loss (*lesion*) at 14 and 147 dpi – IHC NeuN. The graph depicts the untreated mice (yellow) without (no filling) and with neuronal loss (v-pattern) and mice with tamoxifen treatment (blue, dotted) at 14 and 147 dpi. Mice with neuronal loss are additionally marked with a red dot. 7 of 12 untreated mice (58 %) do not show neuronal loss at 14 dpi. 5 of 12 untreated mice (42%) and 6 of 6 mice (100%) with tamoxifen application at 0, 2 and 4 dpi (*Tam0dpi*) are affected by neuronal loss at 14 dpi. Most mice (79-80%) do not show neuronal loss at 147 dpi. 20-21 % of the mice (*NoTam*: 3 of 14; *Tam0dpi*: 1 of 5) still show neuronal loss at 147 dpi. Tamoxifen application had no long-term effect on the reduction of neuronal loss (p= 0.8).
